# Oncosuppressive Role of RUNX3 in Human Astrocytomas

**DOI:** 10.1155/2019/1232434

**Published:** 2019-08-05

**Authors:** Giedrius Steponaitis, Arunas Kazlauskas, Paulina Vaitkienė, Vytenis P. Deltuva, Mykolas Mikuciunas, Daina Skiriutė

**Affiliations:** Laboratory of Molecular Neurooncology, Neuroscience Institute, Lithuanian University of Health Sciences, Eiveniu Str. 4, 50161 Kaunas, Lithuania

## Abstract

**Background:**

Gliomas are the most common and aggressive among primary malignant brain tumours with significant inter- and intratumour heterogeneity in histology, molecular profile, and patient outcome. However, molecular targets that could provide reliable diagnostic and prognostic information on this type of cancer are currently unknown. Recent studies show that certain phenotypes of gliomas such as malignancy, resistance to therapy, and relapses are associated with the epigenetic alterations of tumour-specific genes. Runt-related transcription factor 3 (*RUNX3*) is feasible tumour suppressor gene since its inactivation was shown to be related to carcinogenesis.

**Aim:**

The aim of the study was to elucidate RUNX3 changes in different regulation levels of molecular biology starting from epigenetics to function in particular cases of astrocytic origin tumours of different grade evaluating significance of molecular changes of RUNX3 for patient clinical characteristics as well as evaluate RUNX3 reexpression effect to GBM cells.

**Methods:**

The methylation status and protein expression levels of RUNX3 were measured by methylation-specific PCR and Western blot in 136 and 72 different malignancy grade glioma tissues, respectively. Lipotransfection and MTT were applied for proliferation assessment in U87-MG cells.

**Results:**

We found that* RUNX3* was highly methylated and downregulated in GBM.* RUNX3* promoter methylation was detected in 69.4% of GBM (n=49) as compared to 0 to 17.2% in I-III grade astrocytomas (n=87). Weighty lower RUNX3 protein level was observed in GMB specimens compared to grade II-III astrocytomas. Correlation test revealed a weak but significant link among Runx3 methylation and protein level. Kaplan-Meier analysis showed that increased RUNX3 methylation and low protein level were both associated with shorter patient survival (p<0.05). Reexpression of RUNX3 in U87-MG cells significantly reduced glioma cell viability compared to control transfection.

**Conclusions:**

The results demonstrate that* RUNX3* gene methylation and protein expression downregulation are glioma malignancy dependent and contribute to tumour progression.

## 1. Introduction

Glial brain tumours originating from glial cells are intracranial solid neoplasms. According to classification system of the World Health Organisation (WHO) based on histological evaluation, brain tumour grade is determined by necrotic cells in the centre of the tumour, increased mitotic activity, the presence of nuclear pleomorphism, and angiogenesis. Brain tumour malignancy is associated with tumour heterogeneity, recurrence, patient survival, and therapy response [1]. For instance, grade I tumours, like pilocytic astrocytomas (according to the 4th edition of WHO classification), are curable glial tumours, while grade IV astrocytic tumours, glioblastomas, are rapidly progressive and lethal [2]. In the last years evidences indicating evolvement of epigenetic alterations in cancer development and in the response to therapy steadily accumulated. Although histology in neurooncology represents gold standard in diagnostics, the recently described identification of molecularly different glioblastoma oncotypes and its correlation with clinical characteristics is important step in patient stratification into clinically distinct subgroups that could eventually benefit from personalized therapeutic strategy [3–5].

RUNX3 protein is a transcription factor, containing a highly conserved DNA binding domain which binds to a DNA core motif of 5′ pyGpyGGT 3′ designated as a “runt domain, RD” which shares a sequence similarity with D. melanogaster RUNX. Three different mammalian RUNX protein family members have been identified: RUNX1 (also called PEBP2AB, CBFA2/AML1), RUNX2 (PEBP2aA, CBFA1, AML3), and RUNX3 (PEBP2aC, CBFA3/AML2). All three family proteins physically associate with SMAD proteins, downstream targets of TGF-beta/BMP signalling, and play roles in mammalian development [7]. RUNX3 is the least studied and the least characterized of all RUNX members. During normal development, RUNX3 is found to be expressed in the hematopoietic system, in osteoblasts and chondrocytes and in neurotrophin-positive neurons of the dorsal root ganglia [8] indicating its role in neuronal development [9]. In adult organism RUNX3 expression persists in the hematopoietic system [8]; however, its biological function is largely unexplored. RUNX3 was also noticed to be involved into oncological events. RUNX3 has been variously described as a tumour suppressor or promoter, occasionally with a conflicting result in the same cancer and possible reflecting a complex role of RUNX3 in oncogenesis [10]. The study of loss of RUNX3 expression during progression to invasive gastric cancer compared to the normal gastric epithelium was the first suggesting tumour suppressive function for RUNX3 [11]. Later on, a number of studies meet the same results suggesting gene suppressor role for RUNX3 in other solid tumours such as colon [12], lung [13], breast [14], glioblastoma [15], renal cell [16], hepatocellular carcinomas [17], chondrosarcoma [18], etc. Many other cancer studies based on epigenetics have suggested that RUNX3 downregulation in cancers could be the result of hypermethylation of the promoter of RUNX3 [19–21]. Hypermethylation of RUNX3 promoter was also observed in glioblastoma cell lines and primary tumour tissue compared to normal human brain tissue [15, 22]. Moreover, RUNX3 methylation was considered as a potential biomarker of aggressiveness of gliomas [23].

Nevertheless, other studies showed opposite data and suggested tumour promoting or oncogenic role for RUNX3. High expression of RUNX3 was associated with ovarian cancer [24] as well as with poor histologic differentiation, metastasis, and invasion in head and neck squamous cell carcinoma [25], with pancreatic ductal adenocarcinoma [26] and basal cell carcinoma [27], with childhood acute myeloid leukaemia [28], and with inflammatory breast [29] and gastric cancers [30, 31].

In current study we aimed to evaluate* RUNX3* gene methylation and protein expression in only astrocytic origin tumours of different grade to estimate association between methylation frequency and protein expression, as well as evaluate the RUNX3 alteration effect on patient survival. In the present study for the first time RUNX3 promoter methylation and protein expression was analysed in the same specimens of brain tumour to reveal if the link of RUNX3 methylation and silencing which was shown in glioma cell lines meet the similar processes in astrocytoma specimens.

## 2. Material and Methods

### 2.1. Patients and Tissue Samples

In total 136 postoperative grade I-IV astrocytoma specimens were used for the analysis: 14 (10.3%) grade I pilocytic astrocytomas, 44 (32.4%) grade II diffuse astrocytomas, 29 (21.3%) grade III anaplastic astrocytomas, and 49 (36%) glioblastomas grade IV. All the specimens of astrocytoma were surgically resected from patients without prior treatment (no patients had received chemo- or radiotherapy before surgery) in Neurosurgery Clinics of Hospital (NCH) of Lithuanian University of Health Sciences Kaunas, Lithuania, during the period from 2003 to 2017. Brain tumour tissue specimens after dissection were snap-frozen in liquid nitrogen and stored until analysis. Written informed patient consent was obtained for every patient under the approval of Kaunas Regional Biomedical Research Bioethics Committee. The study was accomplished under the principles of Declaration of Helsinki. The clinical patient data such as age at the time of the tumour resection, gender, time of the last follow-up, were collected for each patient. The survival of the patients was calculated from the date of tumour resection to the date of death of the patient or database closure (September 2018) date if the patient was still alive.

Overall study sample of 136 glioma patients consisted of 44.8% (n=61) males and 55.1% (n=75) females, patient median age was 48 years (range 18-89 years), and median overall survival time after diagnosis was 30.9 months (range 0.2 to 154 months). Patient age does not differ between astrocytoma grade I to III (median age 37.6 years), but patients in those groups were significantly younger compared to glioblastoma group (median age 65 years, Kruskal-Wallis test, p<0.001).

### 2.2. Methylation-Specific PCR

**Figure 1:**
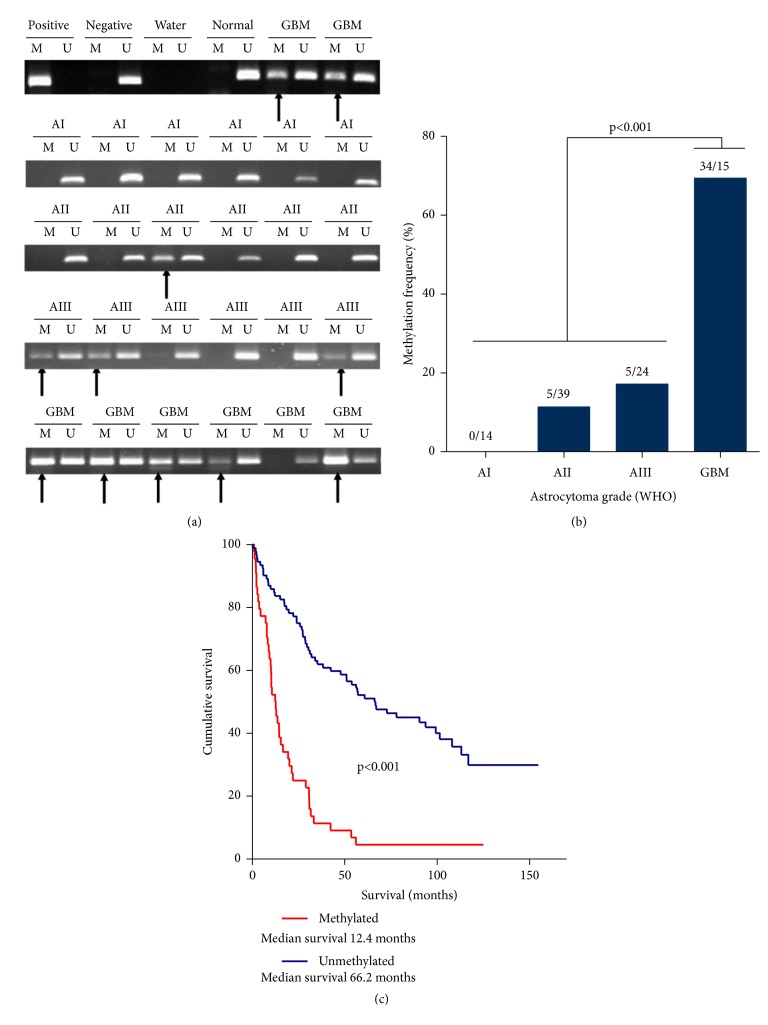
(a) Representative results for methylation-specific PCR of RUNX3 gene in different malignancy grade gliomas. M: PCR with primers for methylated RUNX3. U: PCR with primers for unmethylated RUNX3. Positive control: bisulfite converted universal methylated human DNA standard (Zymo Research, USA). Negative control: bisulfite converted normal lymphocyte DNA. Normal: “normal human brain DNA” (Zymo Research, cat. no. D5018). Arrows indicate methylated allele. (b) RUNX3 promoter methylation frequency in different astrocytoma grade. GBM revealed significant increase in RUNX3 gene promoter methylation frequency compared to I-III WHO grade astrocytomas (p<0.001, chi-square test). (c) Kaplan-Meier curves for survival (months) of all glioma patients (n=136) stratified by RUNX3 gene methylation status revealed significantly shorter survival of patients with RUNX3 methylated tumours (Log-rank test, *χ*^2^=44.68, df=1, p<0.001).

DNA extraction from human brain tumour tissue applying modified salting-out method, DNA bisulfite modification using EpiJET Bisulfite Conversion Kit (Cat No: K1461, Thermo Scientific, Inc.), methylation-specific amplification using hot start polymerase (Cat No: K1052, Thermo Scientific Inc.), and methylation detection procedures were performed as previously described [32]. MSP primers designed and verified by Mueller, 2007, were applied for RUNX3 methylation analysis. MSP primers for methylated allele were 5'-TTACGAGGGGCGGTCGTACGCGGG-3' (sense) and 5'-AAAACGACCGACGCGAACGCCTCC-3' (antisense) and for unmethylated allele: 5'-TTATGAGGGGTGGTTGTATGTGGG-3' (sense) and 5'-AAAACAACCAACACAAACACCTCC-3' (antisense). 10 pmol of each primer in a total volume of 12 *μ*l was used for MSP. MSP was carried out for 38 cycles applying at 95°C for 15 sec. for denaturation, 67°C for 30 sec. for annealing, and 72°C for 15 sec for the extension. The signals of the correct molecular weight of amplified DNA with primers for methylated or unmethylated sequence were registered as a methylated or unmethylated promoter of the gene (Figure 1(a)). In a case of amplification of both variants (methylated and unmethylated), gene promoter of the sample was considered as being methylated.

### 2.3. Whole-Tissue Protein Extract Preparation and Immunoblot Analysis

Preparation of whole-tissue extracts of the tumour specimens, SDS-PAGE, and protein transfer to nitrocellulose membrane procedures was done as previously described [32]. Primary rabbit antibody against RUNX3 (Antibodies-Online, cat no. ABIN739370) diluted 1:500 in 5% nonfat milk in PBS was used for RUNX3 protein detection applying 2-hour incubation on a platform shaker at room temperature. After washing in PBS buffer supplemented with 0.5% of Tween-20, membranes were incubated with the anti-rabbit secondary antibody conjugated with horseradish peroxidase (Life Technologies, cat no. 656120, dilution 1:2000) for 1-hour at room temperature. Signals were visualized using enhanced chemiluminescence reagent Ampliflu™ Red (Sigma-Aldrich, cat no. 90101) and recorded using gel visualization system GelDoc-It®2 imager (Analytik Jena AG). Detection assay of input control, ACTB, on the same membranes after mild stripping and reprobing was performed as previously described [32]. Values of RUNX3 and ACTB signals were calculated applying image analysis program ImageJ version 1.47 (National Institutes of Health, USA).

### 2.4. Cell Culturing and Proliferation Assay

Human glioblastoma cell line U87-MG was used for functional assessment of RUNX3. The U87-MG cell line was purchased from Sigma-Aldrich (source: European Collection of Authenticated Cell Culture, ECACC, cat. no: 89081402). Cells were cultivated in high glucose Dulbecco's Modified Eagle Medium (DMEM) with Phenol Red, “GlutaMAX™ ” (Gibco, cat. no 10566016) supplemented with 10% (v/v) fetal bovine serum (FBS) (Gibco, cat. no 10566016) and 1% (v/v) Penicillin and Streptomycin (P/S). Cells were maintained at 37°C in a humidified incubator containing 5% (v/v) CO2. Cells visualization was accomplished under Etaluma LS620 microscope (Lumascope) applying standard phase contrast microscopy for routine cell visualization and exited microscopy with a green filter (Excitation 473-491 nm, Emission 502-561 nm) for green fluorescent protein (GFP) detection. GFP plasmid (pcDNA4TO-GFP) transfected cell was applied to evaluate transfection efficiency. Expression vector pcDNA3 with RUNX3 gene (pcDNA3-RUNX3) was gifted from PhD Dominic Chih-Cheng Voon, Cancer Science Institute of Singapore, National University of Singapore. Cell viability was monitored applying the MTT assay (Invitrogen, cat. no M6494) in 96-well flat-bottomed microplates after 24-hours after transfection. Approximately 12.500 U87 cells per well were used for transfection with “Lipofectamine™ 3000 Transfection Reagent” (Invitrogen, cat. no. L3000015) and 100 ng of plasmid DNA according to the manufacturer's protocol. Microplates were analysed by the Multiskan™ GO Microplate Spectrophotometer measuring absorption at 550 nm (Formazan absorption) and at 620 nm (background normalization).

### 2.5. Statistical Analysis

Differences across two independent groups were analysed applying Mann-Whitney U test, and Kruskal-Wallis test was used for differences estimation across more than two independent groups. For categorical data sets analysis (such MSP data) chi-square test was applied. The Kaplan-Meier curves method was applied to estimate survival functions and the log-rank test used to compare the difference of survival between groups. Patient survival was calculated from the data of tumour resection to the date of patient death, or database closure date (September 2018). Cox regression model was applied to assess the independence of prognostic factors such as gender, age, and molecular factors such as RUNX3 methylation and protein expression which were first examined individually applying univariate Cox regression analysis, and all factors that had a strong impact on survival were then evaluated jointly in multivariate Cox regression analysis applying Backward Conditional method.

Statistical calculations were performed using GraphPad Prism for Windows (v. 6.0, GraphPad Software, Inc.) and SPSS statistics for Windows (v. 22.0, IBM) software packages. The value of p<0.05 was considered significant.

## 3. Results

### 3.1. RUNX3 Promoter Methylation Frequency Is Gradually Increasing along Astrocytoma Grade

MSP analysis revealed positive* RUNX3* promoter methylation (below in the text:* RUNX3 *methylation) in 44 (32.4%) out 136 of glioma patient tumour specimens (Table 1). Should be noted that* RUNX3* gene promoter was unmethylated in normal brain tissue sample (Zymo Research, cat. no. D5018), Figure 1(a).* RUNX3* promoter methylation analysis showed gradually increasing RUNX3 methylation frequency along astrocytoma grade. No methylation signals in any tumour samples of astrocytoma grade I (0 from 14) was detected (Figure 1(b); Table 1).* RUNX3 *methylation frequency increased to 11.4% and 17.2% in astrocytoma grade II and astrocytoma grade III tumours, respectively. The highest* RUNX3* methylation frequency was observed in glioblastoma tissue specimens and even 34 out of 49 samples were methylated (69.4%). Data analysis revealed significant increase in* RUNX3* methylation frequency in glioblastoma tumours as compared to grade I-III astrocytomas (Chi-square test, p<0.001), Figure 1(c).

### 3.2. RUNX3 Protein Expression Is Reduced in Glioblastomas

**Figure 2:**
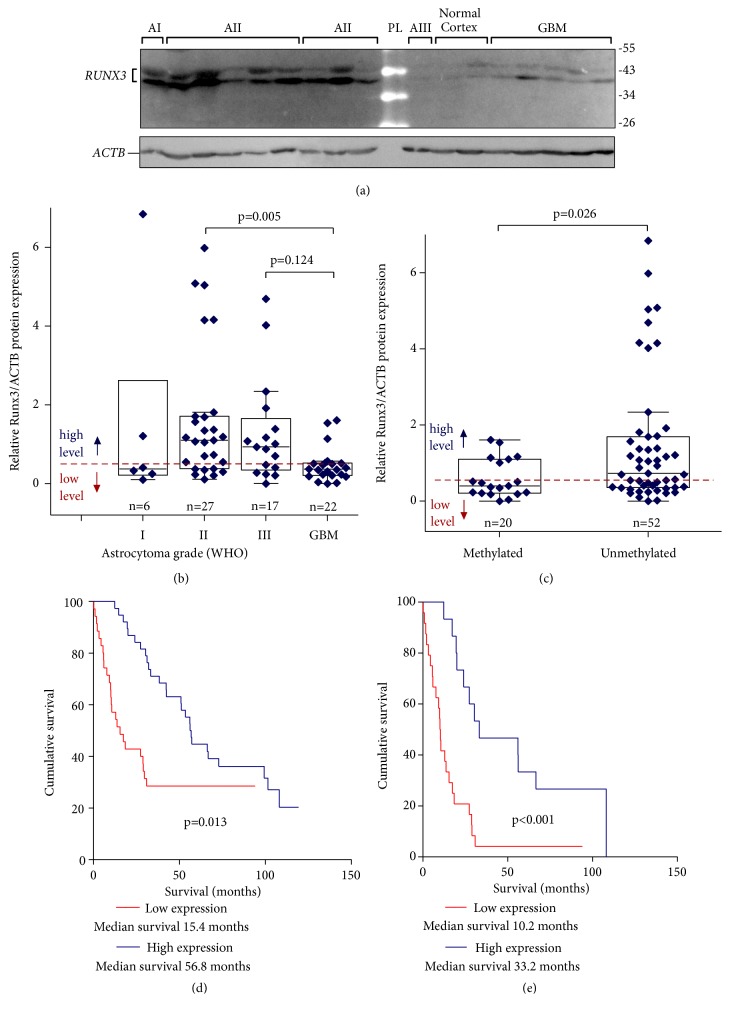
(a) Representative Western blot result of RUNX3 protein expression in astrocytomas. AI-AIII: astrocytoma malignancy grades I-III, respectively; GBM: glioblastoma. Two isoforms of RUNX3 were identified in all the specimens that is consistent with what has been described in the literature [6]. (b) RUNX3 protein expression levels in different astrocytoma grade. Protein expression was significantly downregulated in glioblastomas (GBM) as compared to grade II astrocytomas (p<0.005, Kruskal-Wallis test) and a tendency as compared to grade III astrocytomas (p=0.124, Kruskal-Wallis test). (c) Relative RUNX3 protein expression stratified by promoter methylation groups. Significant association between RUNX3 gene methylation and protein expression was found (p=0.026, Kruskal-Wallis test). (d) Kaplan-Meier curves for survival of all astrocytoma patients (n=72) stratified in two groups (low; high) according to protein expression revealed significant better survival rates for patient with high RUNX3 protein level (Log-rank test, *χ*2=6.11, df=1, p=0.013). (e) Kaplan-Meier survival curves of high malignancy grade (III-IV) astrocytoma patients only (n=39) (Log-rank test, *χ*2=13.74, df=1, p<0.001).

RUNX3 expression evaluation at protein level was performed on the same glioma samples applying Western blot (WB) analysis, and in a total 72 glioma specimens, among which 6 were astrocytoma grade I, 27 grade II, 17 grade III, and 22 grade IV (glioblastoma) were used (Table 1). In tumour specimens RUNX3 protein level showed variable pattern; the expression varied between highly expressed to very weak or not detectable at all, Figure 2(a). RUNX3 revealed significantly lower protein level in glioblastomas compared to grade II tumours (Kruskal-Wallis test, p=0.005) and a tendency of lower GBM RUNX3 protein level compared to grade III astrocytomas (Kruskal-Wallis test, p=0.124), Figure 2(b). RUNX3 protein expression reduction was observed in the majority of studied glioblastoma specimens as compared to lower-grade gliomas.

Significant association between RUNX3 gene methylation and protein expression was found (Kruskal-Wallis test, p=0.026) Figure 2(c). Correlation analysis revealed a weak but significant link among Runx3 methylation and protein level. (Spearman correlation coefficient -0.269, p=0.024, n=72).

### 3.3. Molecular RUNX3 Variations in Tumours Are Associated with Patient Clinicopathological Variables

Promoter methylation of* RUNX3* was closely associated with patient age (Mann-Whitney test, p<0.001), tumour malignancy grade (*χ*^2^ test, p<0.001), and total patient survival (Mann-Whitney test, p<0.001) as well as patient 2-year patient survival after tumour resection (≤24 and >24 months; *χ*^2^ test, p=0.001). Patient gender did not show any importance for RUNX3 methylation in tumours (*χ*^2^ test, p>0.05), Table 1.* RUNX3* methylated gliomas were more likely to be high grade than low grade and gene methylation was associated with older patient age. Patients surviving more than 24 months were more likely having tumours without* RUNX3* gene methylation than patients surviving less than 24 months period. The clinicopathological significance of RUNX3 gene expression at protein level was evaluated by analysing its expression link with clinical parameters. Kruskal-Walis test revealed significant RUNX3 protein level associations with astrocytoma pathological grade (p=0.007), patient 2-year survival after tumour resection (≤24 and >24 months, p=0.001), and patient disease appearance age (≤50 and >50 years; p=0.037), but not gender (p=0.707), Table 1. Glioma patients who survived longer than 2-years more likely have highly RUNX3 expressed tumours than shorter surviving patients. Associations between RUNX3 methylation as well as protein level and patient clinicopathological characteristics are summarized in Table 1.

### 3.4. Astrocytoma Patient Survival Is Associated with RUNX3 Molecular Aberrations

The survival analysis encompassed all the analysed samples irrespective of tumour grade, thus eliminating obscure boundary when separating between grade II and III as well as grade III and IV astrocytomas. All astrocytoma patients were stratified (n=136) in two groups: having tumours with methylated* RUNX3* gene promoter and with unmethylated promoter. Among 136 patients 37 were alive at the end of the study (September 2018) and in statistical analysis were censored. The log-rank test showed that* RUNX3* gene methylation was strongly related to patient survival time (Log-rank test, *χ*^2^=44.68, df=1, p<0.001; Figure 1). Similar results were obtained when analysing high grade tumours (grades III-IV) only (Log-rank test, *χ*^2^=8.74, df=1, p=0.003, n=78; data not shown. More specifically, the patient median survival time in the group with unmethylated gene promoter was 66.17 months [95% CI 34.76-97.58], whereas the median survival time of those with methylated promoter was only 12.35 months [95% CI 8.69-16.02], Figure 1(b). The cumulative 3-year survival rate was 61.95% in RUNX3 unmethylated group, whereas in methylated gene group survival rate was only 11.62%.

Next, we analysed RUNX3 protein expression effect on glioma patient survival. For the purpose we stratified RUNX3 protein expression data into two expression groups based on ROC curve analysis (selecting 2-year overall survival as a state variable). The value of 0.54 of Relative Runx3/ACTB protein expression was selected as cut-off point. Among the 72 glioma specimen, low RUNX3 protein level was assigned for 48.6% (35 of 72), and 51.4% (37 of 72) of samples was assigned for high RUNX3 protein level group, Table 1. Among 72 patients 21 were alive at the end of the study and were censored in the analysis. Survival analysis showed significantly higher survival rates of astrocytoma patients having tumours with high RUNX3 protein level when analysing whole sample set (Log-rank test, *χ*^2^=6.11, df=1, p=0.013, Figure 2(d)) and even stronger connection when analysing malignant astrocytomas (grades III-IV) only (Log-rank test, *χ*^2^=13.74, df=1, p<0.001, Figure 2(e)). The median survival of patients with high RUNX3 protein expression reached 56.77 months [95% CI 41.81-71.74] while patient median survival with low RUNX3 levels reached only 15.37 months [95% CI 6.24-24.52], Figure 2(d).

To evaluate the independence of analysed molecular prognostic factors such as RUNX3 methylation and RUNX3 protein expression, the multivariate Cox regression analysis applying* Backward Conditional* method was performed combining patient age as a clinical covariate (patient gender was not associated with death risk, p>0.05). Multivariate analysis revealed that RUNX3 protein level and patient age but not RUNX3 methylation (p>0.05) were an independent indicators increasing the risk of patient death. Low RUNX3 protein level increases event risk by 1,33-fold (95%CI 1.61-1.08; p=0.004) while older patient age at disease appearance increases event risk by 1.07-fold (95%CI 1.04-1.09; p<0.001), Table 2.

### 3.5. Reexpression of RUNX3 in Glioblastoma U87-MG Cell Line Decreased Cell Viability

**Figure 3:**
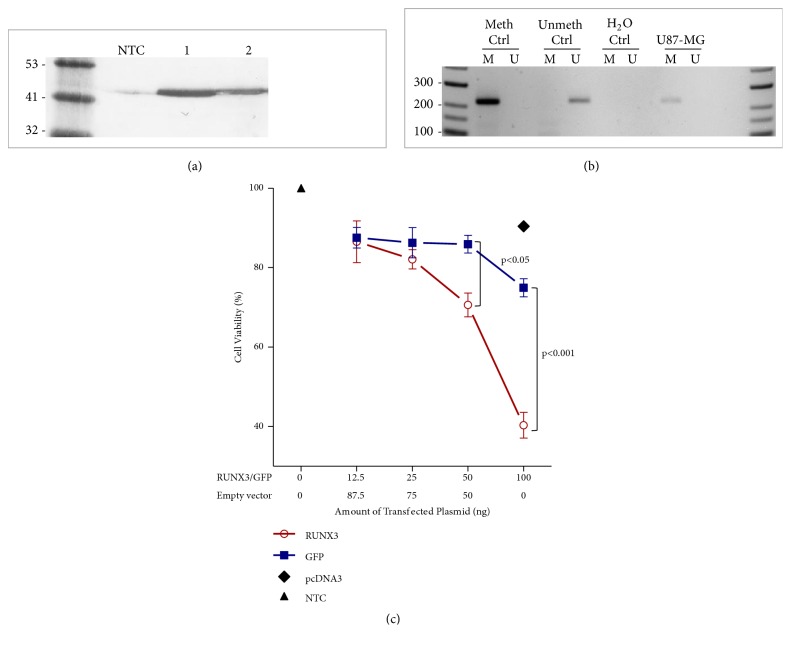
(a) WB of RUNX3 protein expression after U87-MG cells transfection with pcDNA3-RUNX3 construct. NTC: nontransfected cells lysate; 1: cells transfected with 100 ng of pcDNA3-RUNX3 vector; 2: cells transfected with 50 ng of pcDNA3-RUNX3 vector. Equal amount of total protein extract (60 *μ*g) was loaded per each gel lane. The same amount of cell (b) RUNX3 promoter methylation status in U87-MG cells. (c) Results from glioblastoma U87-MG cells viability assay applying MTT test after 24 h of 100 ng of DNA transfection. RUNX3: RUNX3 gene in pcDNA3 expression vector (pcDNA3-RUNX3); GFP: green fluorescing protein in pcDNA4TO expression vector (pcDNA4TO-GFP); pcDNA3: empty (control) vector; NTC: nontransfected cell control. Overexpression of RUNX3 significantly decreased U87-MG cell viability starting from 50 ng of vector used as compared to the cells transfected with GFP vector. Moreover even bigger effect of decreased cell viability was obtained when 100 ng of RUNX3 was transfected.

Since protein expression of RUNX3 is decreased in glioblastomas compared to lower-grade astrocytoma tumours we performed U87-MG cells proliferation analysis after RUNX3 reexpression. U87-MG cells were transfected with pcDNA3-RUNX3, pcDNA4TO-GFP, and empty pcDNA3 (control) plasmids. Transfection efficiency varied between 60 and 75%. 24-hours after transfection cells were subjected to cell proliferation, MTT assay. WB analysis showed that native U87-MG RUNX3 protein expression is barely detectable while RUNX3 promoter is fully methylated, Figures 3(a) and 3(b). Reexpression of RUNX3 in U87-MG cells significantly decreased cells viability as compared to control cells transfected with empty plasmid or plasmid with GFP (p<0.001, when 100 ng of plasmid used), Figure 3(c). Cell viability decreased to 40.2% using 100 ng of RUNX3 construct for transfection compared to nontransfected cell control (NTC), Figure 3(c).

## 4. Discussion

In present analysis we demonstrated that RUNX3 is starting to be deregulated from very onset of gliomagenesis at both epigenetic (methylation) and functional (protein expression) levels and these changes are tightly associated with patient age and survival as well as tumour pathological grade. Functional assessment revealed putative-oncosupressive acting of RUNX3 in astrocytomas since gene is methylated and silenced in GBM cell lines and restoration of RUNX3 expression weighty reduced tumour cell viability.

RUNX3 was first suggested to be a tumour suppressor in gastric cancer. The gastric mucosa of RUNX3 knock-out mouse exhibited hyperplasia due to stimulated proliferation and suppressed apoptosis of epithelial cells which showed resistance to the growth-inhibitory and apoptosis inducing action of TGF-*β* [11]. Since the discovery of the potential role of RUNX3 in gastric cancer,* RUNX3* has been found to be inactivated in various cancers, including colorectal, liver, lung, prostate, and breast as well as gliomas [12–15, 17, 18, 21, 33–35]. Few cancer epigenetic studies have suggested that RUNX3 downregulation could be the result of hypermethylation of the promoter of RUNX3 [15, 19, 20]. Mueller with colleagues were first to show that RUNX3 is hypermethylated in glioblastoma cell lines and primary glioma tumour tissue cells compared to normal human brain tissue. Moreover, they suggested that RUNX3 expression is regulated by promoter methylation since increased mRNA levels of RUNX3 following 5-aza-dC treatment were found in glioma U87 cells [15]. Nevertheless, low sample numbers and the lack of information about RUNX3 alterations in lower-grade gliomas decided the appearance of wider RUNX3 analysis in sample number-rich glioma studies. Mei and colleagues showed that RUNX3 protein expression is decreased in benign and malignant brain tumours as compared to normal or adjacent tissue. Nevertheless they did not found any associations between RUNX3 protein level and patient clinicopathological data [35]. In present study we found tight RUNX3 association with astrocytoma tumour grade as well as patient age and survival. We found that RUNX3 protein expression is reduced in glioblastomas as compared to grade II-III tumours and this reduction is associated with patient overall survival. Since Mei and colleagues combined very diverse origin brain tumours encompassing astrocytomas, ependymomas, and oligodendrogliomas in one analysis, they might have vanished very specific features of tumours of a particular origin. Current analysis revealed that grade I pilocytic astrocytomas (PA) showed different RUNX3 expression profile from other low grade tumours indicating possible distinct molecular features that operate at the onset of PA, since these tumours are the most common benign neoplasms in children or young adults [36]. Nevertheless, the result from both studies indicates that RUNX3 is important player from very beginning of tumorigenesis. Majchrzak-Celińska and colleagues showed RUNX3 promoter methylation changes in different grade gliomas where methylation frequency was consistently increasing along with glioma grade, 0%; 22%; 52%; 62.5%, grades I; II; III; IV, respectively. They also showed significant association between RUNX3 methylation and patients age as well as tumour grade [23]. Very similar data was showed in present study. Besides RUNX3 methylation association with tumour grade and patient age we found very strong association between RUNX3 methylation and patient survival what indicates prognostic importance of RUNX3. Similarly to our results, Saraiva-Esperon and colleagues showed that RUNX3 promoter methylation was associated with poorer patient survival [22]. Another study of RUNX3 methylation encompassing relatively small numbers of specimens (grades II-III-IV, 3-3-12 samples, respectively) failed to find any weighty tumour grade and methylation association; nevertheless they showed significant link between RUNX3 methylation and mRNA expression [37]. Functional study of RUNX3 revealed that reexpression of RUNX3 weighty reduces U87-MG glioblastoma cell viability indicating oncosupressive features of RUNX3. Similar data was shown by Mei et al. when restoration of RUNX3 significantly inhibited U87 and U251 cell invasion and migration abilities [35].

In conclusion, our study revealed that RUNX3 gene methylation frequency is increasing during gliomagenesis, while RUNX3 protein expression is significantly decreasing along with astrocytic origin tumours of different grade and such alterations are tightly associated with patient clinicopathological features. Functional assessment revealed putative-oncosupressive acting of RUNX3 in astrocytomas what is in the line with expression data from astrocytoma specimen's analysis. Significant impact of RUNX3 on patient survival as well as other clinicopathological features indicates gene as potential prognostic marker in astrocytomas.

## Figures and Tables

**Table 1:** Associations between grade I-IV astrocytoma patients' clinical data and RUNX3 gene molecular properties.

	Methylation			Protein expression		
Variable	Unmethylated % (n)	Methylated % (n)	*P* *(χ*^*2*^*; df)*^†^	Low % (n)	High % (n)	*P*
Overall	67.6 (92)	32.4 (44)		48.6 (35)	51.4 (37)	

Grade						
I	100 (14)	0 (0)	**<0.001** *(49.3; 3)*	66.7 (4)	33.3 (2)	**0.007** ^§^
II	88.6 (39)	11.4 (5)		25.9 (7)	74.1 (20)	
III	82.8 (24)	17.2 (5)		35.3 (6)	64.7 (11)	
IV (GBM)	30.6 (15)	69.4 (34)		81.8 (18)	18.2 (4)	

Gender						
Male	65.6 (40)	34.4 (21)	0.641 *(0.22; 1)*	48.8 (20)	51.2 (21)	0.707^*♯*^
Female	69.3 (52)	30.7 (23)		48.4 (15)	51.6 (16)	

Age, years						
<50	88.7 (63)	11.3 (8)	**<0.001** *(30.2; 1))*	32.5 (13)	67.5 (27)	**0.037** ^*♯*^
≥50	44.6 (29)	55.4 (36)		68.8 (22)	31.2 (10)	

Survival, months						
<24	40 (22)	60 (33)	**<0.001** *(32.2; 1)*	80 (20)	20 (5)	**0.001** ^*♯*^
≥24	86.4 (70)	13.6 (11)		31.9 (15)	68.1 (32)	

Methylation						
Unmethylated				39.2 (20)	60.8 (31)	**0.026** ^*♯*^
Methylated				68.4 (13)	31.6 (6)	

^†^P-value estimated by Pearson Chi-square (*χ*^2^) test.

^§^Kruskal-Wallis test.

^*♯*^Mann-Whitney U test.

**Table 2:** Multivariate Cox regression analysis applying Backward Conditional method.

Step	Factor	Multivariate analysis		
		HR	95% CI	*P*-value
1	Patient Age	1.058	1.034-1.083	<0.001
	Runx3 methylation	1.579	0.755-3.3	0.225
	Runx3 protein level	0.777	0.633-0.953	0.015

2	Patient Age	1.065	1.044-1.087	<0.001
	Runx3 protein level	0.752	0.62-0.913	0.004

## Data Availability

The data used to support the findings of this study are available from the corresponding author upon request.
